# Coordination of glioblastoma cell motility by PKCι

**DOI:** 10.1186/1476-4598-9-233

**Published:** 2010-09-03

**Authors:** R Mitchell Baldwin, Gordon M Barrett, Doris AE Parolin, Jana K Gillies, Judith A Paget, Sylvie J Lavictoire, Douglas A Gray, Ian AJ Lorimer

**Affiliations:** 1 Centre for Cancer Therapeutics, Ottawa Hospital Research Institute, 501 Smyth Road, Ottawa, K1H 8L6, Canada; 2 Department of Biochemistry, Microbiology and Immunology, University of Ottawa, Ottawa, Ontario, Canada; 3 Department of Medicine, University of Ottawa, Ottawa, Ontario, Canada

## Abstract

**Background:**

Glioblastoma is one of the deadliest forms of cancer, in part because of its highly invasive nature. The tumor suppressor PTEN is frequently mutated in glioblastoma and is known to contribute to the invasive phenotype. However the downstream events that promote invasion are not fully understood. PTEN loss leads to activation of the atypical protein kinase C, PKCι. We have previously shown that PKCι is required for glioblastoma cell invasion, primarily by enhancing cell motility. Here we have used time-lapse videomicroscopy to more precisely define the role of PKCι in glioblastoma.

**Results:**

Glioblastoma cells in which PKCι was either depleted by shRNA or inhibited pharmacologically were unable to coordinate the formation of a single leading edge lamellipod. Instead, some cells generated multiple small, short-lived protrusions while others generated a diffuse leading edge that formed around the entire circumference of the cell. Confocal microscopy showed that this behavior was associated with altered behavior of the cytoskeletal protein Lgl, which is known to be inactivated by PKCι phosphorylation. Lgl in control cells localized to the lamellipod leading edge and did not associate with its binding partner non-muscle myosin II, consistent with it being in an inactive state. In PKCι-depleted cells, Lgl was concentrated at multiple sites at the periphery of the cell and remained in association with non-muscle myosin II. Videomicroscopy also identified a novel role for PKCι in the cell cycle. Cells in which PKCι was either depleted by shRNA or inhibited pharmacologically entered mitosis normally, but showed marked delays in completing mitosis.

**Conclusions:**

PKCι promotes glioblastoma motility by coordinating the formation of a single leading edge lamellipod and has a role in remodeling the cytoskeleton at the lamellipod leading edge, promoting the dissociation of Lgl from non-muscle myosin II. In addition PKCι is required for the transition of glioblastoma cells through mitosis. PKCι therefore has a role in both glioblastoma invasion and proliferation, two key aspects in the malignant nature of this disease.

## Introduction

Glioblastoma multiforme is a primary brain tumor with a very poor prognosis. Despite the use of aggressive therapeutic approaches combining surgery, radiation and chemotherapy, the median survival time for patients is only 12-14 months [1]. The highly invasive nature of glioblastoma cells blurs tumor margins, making complete surgical resection impossible. Additionally, it is thought that invading cells may be more resistant to radiation and chemotherapy [2]. Inhibition of cell invasion may therefore be an effective strategy to improve the treatment of glioblastoma.

Glioblastoma cell invasion requires that cells have enhanced motility, along with an ability to degrade local tissue barriers. The phosphoinositide 3-kinase (PI 3-kinase) pathway is often constitutively active in glioblastoma as a result of mutations in *PTEN*, as well as mutation and amplification of the epidermal growth factor receptor [3]. These genetic alterations have been shown to promote motility and invasion of glioblastoma cells [4,5]. The PI 3-kinase pathway can activate multiple downstream effectors including the atypical protein kinase C family member PKCι [6,7]. The importance of PKCι as a downstream effector in the PI 3-kinase pathway is emphasized by the fact that PKCι can function as an oncogene in several tumor types [8-10]. On this basis it has been proposed that PKCι is a promising new target for cancer therapy [11].

The activation of PKCι involves direct phosphorylation by phosphoinositide-dependent kinase 1 and association with Cdc42, a small GTPase that is extensively involved in cell migration [6,7,12,13]. The atypical PKCs (PKCι and PKCζ) have been shown to play a role in the establishment of multiple forms of cell polarity, including asymmetric cell division and apical-basal polarity [14]. They form a conserved polarity complex with the scaffold protein, Par-6, that links the atypical PKCs to other proteins including Cdc42, Par-3 and Lgl [15].

We have shown previously that PKCι promotes motility and invasion of glioblastoma cells [16]. PKCι has also been shown to promote the invasiveness of lung cancer cells [17]. These studies have given insight into the role of PKCι in cellular motility and invasion; however they have relied on static analyses of invasion, and did not define precisely the role of PKCι in the dynamic process of cancer cell migration. In this study, we have investigated the role that PKCι plays in the regulation of glioblastoma cell motility using time-lapse videomicroscopy. This showed that PKCι has a critical role in coordinating lamellipod leading edge formation, an essential step in glioblastoma invasion. Interestingly, videomicroscopy also revealed a role for PKCι in mitosis, indicating an additional role for PKCι in the malignant phenotype of glioblastoma.

## Results

### Downregulation of PKCι expression by shRNA

**Figure 1:**
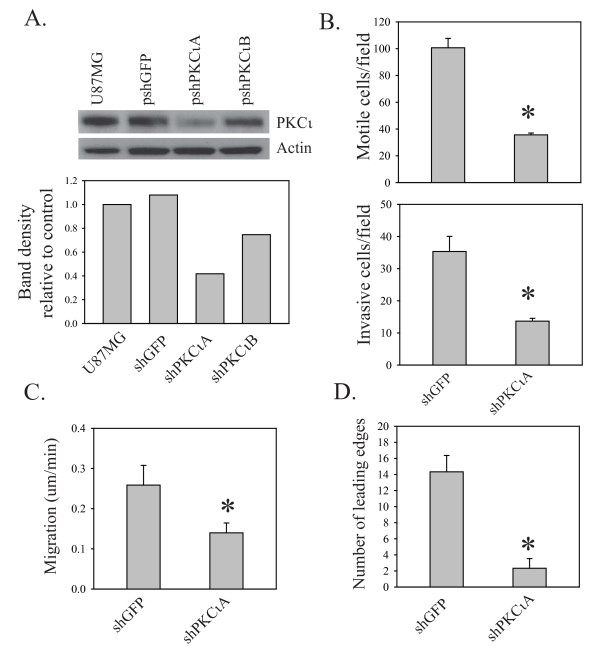
**Effects of stable PKCι depletion on cell motility**. **A**. Western blot analysis and densitometry of PKCι expression in U87MG cells after transduction with retroviral vectors expressing shGFP, shPKCιA and shPKCιB followed by puromycin selection. **B**. Cell motility in U87MG/shGFP and U87MG/shPKCι cells was assessed using Transwell chambers without Matrigel (top) and invasion was assessed using Transwell chambers with Matrigel (bottom). Data are from one of two independent experiments that gave similar results. **C**. Quantitation of motility from videomicroscopy. Migration distance per minute (um/min) was measured using Ziess LSM image browser software. Bar graphs show the mean +/- SD from three independent videomicroscopy experiments for each cell type (mean of 10 cells analyzed per experiment). **D**. Quantitation of leading edge formation from videomicroscopy. Cells from three independent movies were analyzed for the formation of a single dominant leading edge as described in Materials and Methods. Bar graphs show the mean +/- SD.

To stably deplete PKCι in glioblastoma cells, two unrelated PKCι-targeting shRNA expression plasmids (pshPKCιA and pshPKCιB, sequences shown in Additional file 1, Figure S1A) were prepared and expressed in human glioblastoma cell lines using retroviral transduction, along with a GFP-targeting shRNA expression plasmid which was used as a control. Stable pools of transduced glioblastoma cells were isolated following one week of selection in puromycin. In U87MG cells expressing pshPKCιA, PKCι protein expression was reduced by 60% (Figure 1A). pshPKCιB was less efficient in repressing PKCι protein expression, reducing it by only 25% and was not used in further experiments.

The motility and invasive properties of U87MG cells transduced with pshPKCιA were assessed using Transwell chambers. To examine cell motility, control and PKCι-depleted U87MG cells were seeded at the same density in Transwell chambers and 22 h later the number of cells that crossed through the chamber were counted. Stable depletion of PKCι resulted in a 65% decrease in the number of cells that crossed through the chamber (Figure 1B, top). To assess the effects on invasion, control and PKCι-depleted U87MG cells were seeded at equal densities into Transwell chambers that were coated with a Matrigel layer. PKCι depletion also caused a significant reduction (61%) in the number of cells that were able to pass through the Matrigel-coated chambers (Figure 1B, bottom). The fact that the differences in the number of cells that crossed through the chamber in the presence or absence of Matrigel are similar indicates that PKCι affects the invasion of glioblastoma cells primarily by promoting cell motility. This is the same phenotype that we described previously with transient transfection of two different RNA duplexes targeting PKCι [16].

### Time-lapse videomicroscopy of cell motility in U87MG cells stably depleted of PKCι

**Figure 2:**
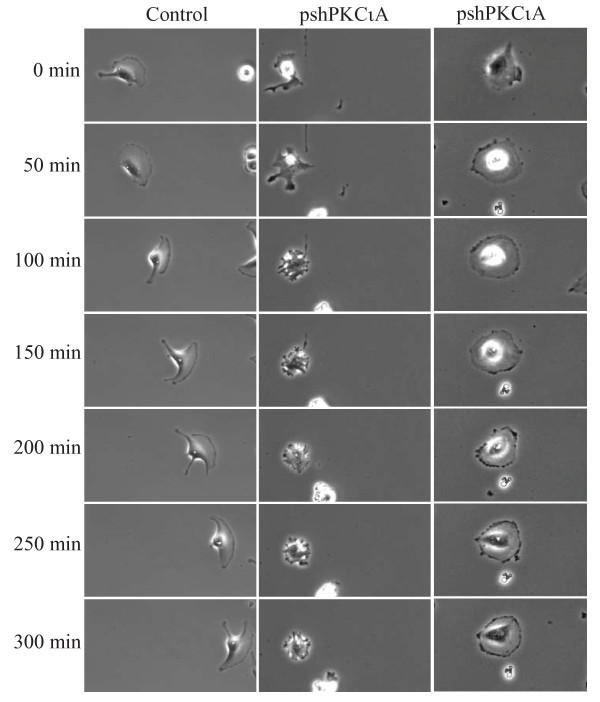
**Examples of motility defects assessed using time-lapse videomicroscopy**. Representative examples comparing cell motility in control and PKCι-depleted cells. Still images from videomicroscopy are shown, with times indicated on the left. Representative full videos are shown in Supplementary videos S1 (control) and S2 (PKCι-depleted).

To gain more insight into the mechanism by which PKCι promotes glioblastoma cell motility, videomicroscopy was used. Control and PKCι depleted U87MG cells were plated into a live cell imaging plate at a density of 10^3 ^cells to allow space for migration. Phase contrast images of the cells were taken at 5 min intervals for 20 h and compiled to generate a time-lapse video. Quantitation of cell movement from videomicroscopy images confirmed the impairment in cell motility in PKCι-depleted cells. Overall migration rates were determined using data from three independent time-lapse videos of each cell line. PKCι-depleted cells had a 45% reduction in migration distance per minute (0.14 μm/min compared to 0.26 μm/min in control cells (Figure 1C)). Quantitation of lamellipod leading edge formation from videomicroscopy showed that cells depleted of PKCι had a markedly impaired ability to form a single, dominant leading edge (Figure 1D). Videomicroscopy showed that the loss of motility was not due to a simple shutdown of cell movement, but instead was due to a loss of coordination in this process. Control cells show an initial extension of a single leading process, followed by translocation of the nucleus toward the lagging edge and finally retraction of the trailing process (Figure 2 and Additional file 2, Video 1). This results in the substantial movement of cells before they change direction. In contrast, PKCι-depleted cells primarily generate multiple short protrusions that emanate from all sides of the cell (Figure 2 and Additional file 3, Video 2); a smaller number of cells show a flattened appearance which appears to be due to an attempt to form a leading edge around the entire circumference of the cell (Figure 2 and Additional file 3, Video 2). The consequence of this is that there is little or no net movement of cells.

### Inhibition of PKCι activity using an atypical PKC specific pseudosubstrate peptide impairs cell motility

**Figure 3:**
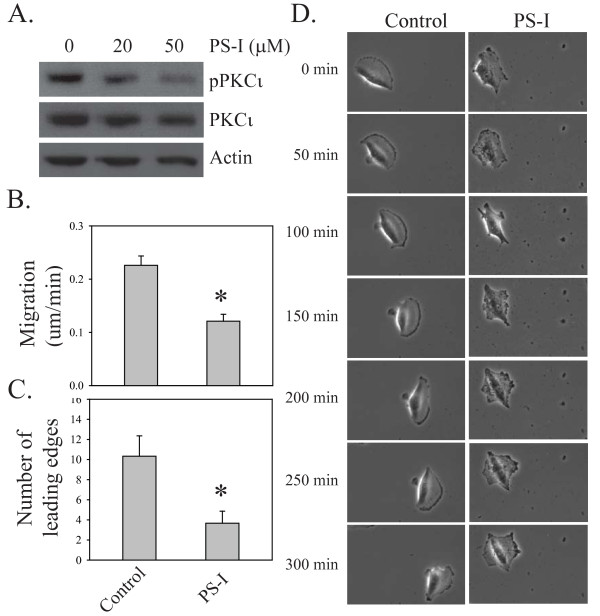
**Effects of PKCι pseudosubstrate inhibitor peptide on cell motility**. **A**. U87MG cells were treated with 20 or 50 μM atypical PKC pseudosubstrate peptide inhibitor (PSI) for 2 h. Whole cell lysates were then analyzed for levels of phosphorylated PKCι and total PKCι by Western blotting. **B**. Migration distance per minute (um/min) was quantitated using Ziess LSM image browser software. Bar graphs show the mean +/- SD from three independent videomicroscopy experiments each for control and PS-I treated U87MG cells (mean of 10 cells quantified per experiment). **C**. Quantitation of leading edge formation from videomicroscopy. Cells from three independent movies were analyzed for the formation of a single dominant leading edge. Bar graphs show the mean +/- SD. **D**. Representative examples comparing cell motility in control cells and cells treated with 20 μM PS-I. Still images from videomicroscopy are shown, with times indicated on the left. A representative full video is shown in Supplementary video S3.

As a second method to assess the role of PKCι in glioblastoma cell motility, U87MG cells were treated with an atypical PKC specific inhibitor (PS-I). This inhibitor is an atypical PKC pseudosubstrate that has been myristoylated to make it cell permeable. Although it inhibits both members of the atypical PKC family (ζ and ι), we have shown previously that U87MG cells only express PKCι, so that we can ascribe any effects of this peptide specifically to inhibition of PKCι (see also Additional file 1) [18]. Treatment of U87MG cells with 20 or 50 μM of PS-I caused a reduction in PKCι activity as assessed by phosphorylation at threonine 555 (Figure 3A). Quantification of the migration rate by videomicroscopy showed that PS-I treatment caused a 47% reduction in migration distance per minute (0.12 μm/min) compared to control cells (0.23 μm/min; Figure 3B). Consistent with the shPKCιA expressing cells, U87MG cells treated with PS-I showed an impaired ability to generate a single dominant leading edge (Figure 3C), with most cells forming multiple small protrusions instead (Figure 3D and Additional file 4, Video 3). A172 human glioblastoma cells in which PKCι was either depleted with shRNA or inhibited with PS-I also showed decreased motility when analyzed by videomicroscopy, although the effect was less marked as these cells are less motile than U87MG cells (Additional file 5, Figure S2A).

### Effects of PKCι on Lgl in migrating glioblastoma cells

**Figure 4:**
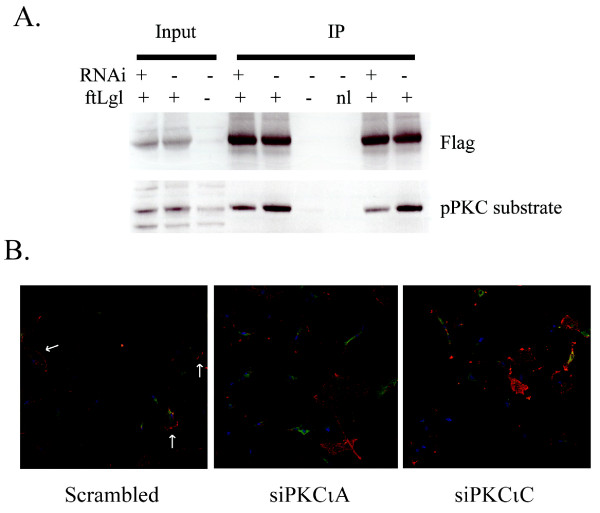
**Effects of PKCι depletion on Lgl**. **A**. U87MG cells expressing either empty vector (-) or vector expressing Flag-tagged Lgl (+) were either mock-transfected (-) or transfected with an RNA duplex A targeting PKCι (+). Three days after transfection, cells were lysed and Flag-tagged Lgl was immunoprecipitated. Immunoprecipitates were solubilized in Laemmli buffer, electrophoresed on SDS-PAGE gels and transferred to membranes for analysis by Western blotting. Membranes were probed with antibodies to Flag epitope or phosphorylated PKC substrate, as indicated. In the lane marked "nl", the immunoprecipitation was performed with no lysate (buffer only). **B**. U87MG/Lgl cells were grown on gelatin-coated coverslips and transfected with either scrambled duplex, RNA duplex A targeting PKCι or RNA duplex C targeting PKCι. Two days later cells were fixed and immunocytochemistry for Flag-tagged Lgl (red) and non-muscle myosin IIA (green) was performed. Nuclei were stained with DAPI.

Lgl is a cytoskeletal protein that has been shown to be a direct substrate for phosphorylation by PKCι [19,20]. This phosphorylation inactivates Lgl, disrupting its ability to interact with cell membrane and non-muscle myosin II [21]. We screened a number of antibodies to Lgl, but did not find one that reliably detected endogenous Lgl in glioblastoma cells without recognizing additional species on Western blots. To overcome this problem, we cloned human Lgl, added an amino-terminal Flag epitope sequence, and transduced this into U87MG cells. U87MG cells expressing Lgl cell showed similar rates of proliferation compared to normal U87MG cells (data not shown) and exhibited similar abilities to form a coordinated leading edge based on microscopy assessment (see Figure 4B). To assess Lgl phosphorylation by PKCι in glioblastoma cells, Flag-tagged Lgl was immunoprecipitated from cells with and without PKCι depletion. Immunoprecipitated Lgl was then analyzed by Western blotting using antibody to PKC consensus phosphorylation site. Lgl was constitutively phosphorylated in glioblastoma cells and this was reduced with PKCι depletion (Figure 4A). PKCι therefore constitutively inactivates Lgl in U87MG cells. We also attempted to generate U87MG cells expressing a mutant, non-phosphorylatable Lgl in which five hinge region serines were mutated to alanine. However we were unable to isolate populations expressing non-phosphorylatable Lgl after retroviral transduction, suggesting that Lgl phosphorylation is required for normal growth of U87MG cells.

**Figure 5:**
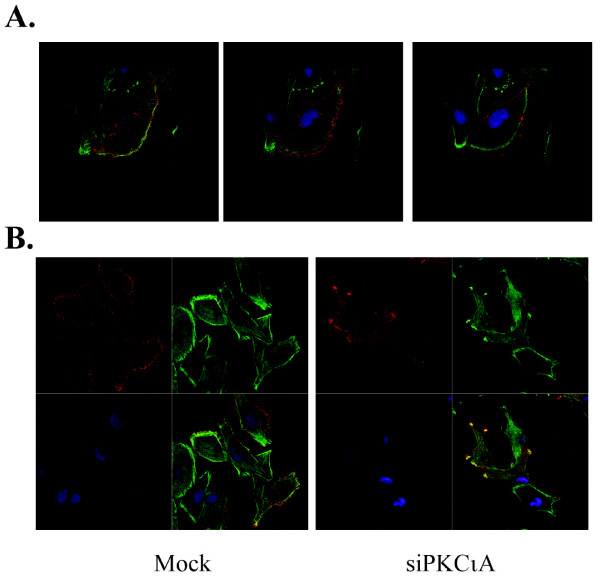
**Confocal microscopy of Lgl and non-muscle myosin IIA in PKCι-depleted cells**. **A**. Immunocytochemistry for Flag-tagged Lgl (red) and non-muscle myosin IIA (green) was performed on U87MG cells transduced with Flag epitope-tagged Lgl cDNA. Nuclei were stained with DAPI (blue). Three serial confocal optical sections are shown for a cell with a distinct leading edge, with the section closest to the substratum on the left. **B**. Confocal images of U87MG cells transduced with Flag epitope-tagged Lgl cDNA that were either mock-transfected (left) or transfected with an RNA duplex targeting PKCι (right). Flag-Lgl (red), non-muscle myosin IIA (green) and DAPI (blue) images are shown separately and as a merged image in the bottom right quadrant.

In U87MG/Lgl cells that showed formation of a lamellipod, Lgl was concentrated at the leading edge of the lamellipod, in agreement with a previous study (Figure 4B, left panel)[20]. A detailed analysis by confocal microscopy showed that Lgl did not colocalize with non-muscle myosin IIA at the leading edge (Figure 5A). Instead, Lgl was concentrated in a band in front of non-muscle myosin IIA. This is in agreement with previous data that non-muscle myosin IIA is excluded from the lamellipod leading edge [22]. When flag-tagged Lgl-expressing cells were depleted of PKCι, this pattern was changed (Figure 4B). Cells showed the presence of multiple sites in which Lgl was concentrated and confocal microscopy showed that Lgl colocalized with non-muscle myosin II at these sites (Figure 5B). This is consistent with a model in which Lgl is recruited to nascent leading edges and is inactivated there by PKCι during leading edge maturation.

### Impaired cell division in U87MG cells depleted of PKCι

**Figure 6:**
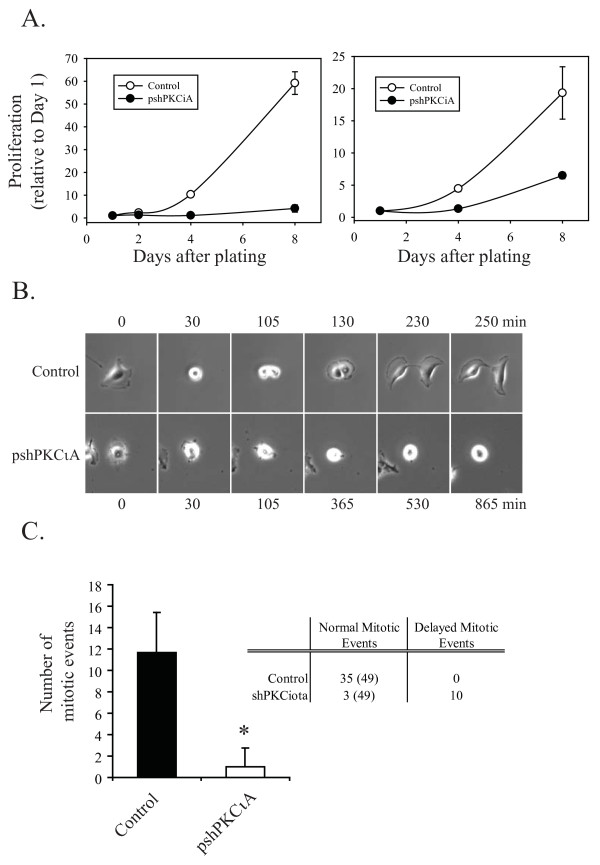
**Effects of stable PKCι depletion on proliferation**. **A**. Control cells or cells expressing pshPKCιA were plated at equal densities and viable cell numbers using trypan blue exclusion were counted at days 1, 2, 4 and 8 after plating. Left panel, U87MG cells; right panel, A172 cells. **B**. Representative example of a control and PKCι depleted U87MG cell undergoing mitosis. Still images from videomicroscopy are shown, with times indicated. A full video is shown in Supplementary video S2. **C**. Quantitation of the number of mitotic events observed during live cell image analysis (20 h) for control U87MG cells or pshPKCιA expressing cells. Data are the mean +/- SD of three independent live cell experiments for each cell line. Inset table indicates the number of normal mitotic events (total starting cell numbers from the three experiments in parentheses) and the number of abnormal, delayed mitotic events observed over the 20 h analysis for control and PKCι-depleted U87MG cells.

The effects of stable PKCι depletion on glioblastoma cell proliferation was evaluated by counting cells using trypan blue exclusion to distinguish live from dead cells. Viable cell numbers were determined 1, 2, 4 and 8 days following plating of control and PKCι depleted cells at equal densities. Stable depletion of PKCι in both U87MG and A172 human glioblastoma cells significantly reduced growth rate compared to control cells (Figure 6A). This was also seen in U87MG and A172 cells transiently transfected with two different RNA duplexes targeting PKCι (Additional file 5, Figure S2B). Time lapse videomicroscopy also confirmed that PKCι-depleted cells had reduced proliferation and showed that this was due, at least in part, to impaired mitosis (Figure 6B and 6C and Additional file 3, Video 2). At mitosis, U87MG cells undergo the rapid cell rounding that has been described for many other cell types. They then divide, flatten out and reinitiate cell movement. This process takes from 150 to 300 min (Figure 6B). In three independent videomicroscopy experiments, the mean number of complete mitotic events observed over 20 h averaged 12 per field of view (Figure 6C inset table). The total number of complete mitotic events observed in the three control experiments was 35; these all occurred within the 150-300 minute time frame and were therefore considered "normal" mitotic events. In PKCι-depleted U87MG cells, the mean number of complete mitotic events observed over 20 h was 1 per field of view for three independent experiments (starting cell densities were similar for both cell types). Two factors contributed to this reduction. The first is a reduction in the number of cells that enter mitosis. The second factor is that the majority of cells that entered mitosis remained rounded for > 500 min, with many failing to complete mitosis within the 20 h timeframe of the videos. In PKCι-depleted cells a total of 10 delayed mitotic events were observed in three separate experiments and only 3 normal events (Figure 6C inset table).

**Figure 7:**
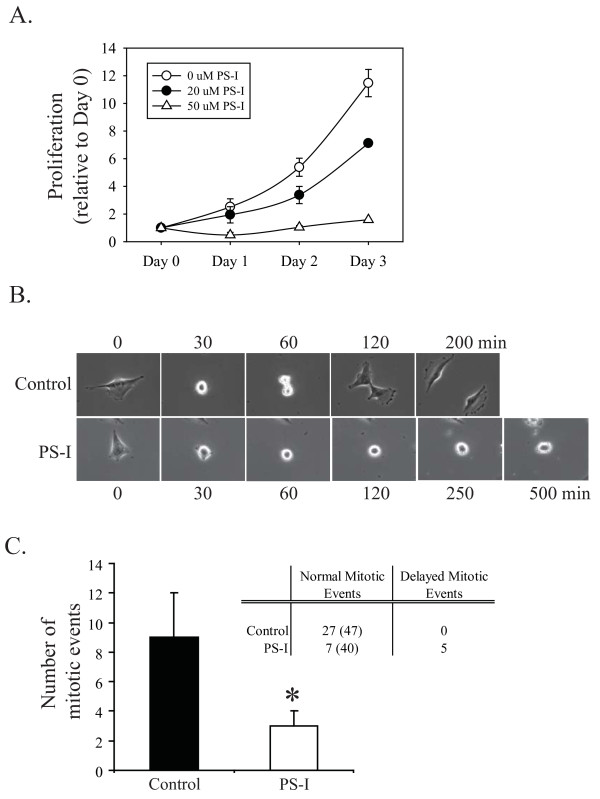
**Effects of PKCι pseudosubstrate inhibitor peptide on cell proliferation**. **A**. U87MG cells were plated at equal densities and 24 h later either control treated or treated with 20 or 50 μM PS-I and viable cell numbers were determined using trypan blue exclusion at days 0, 1, 2 and 3 after treatment. **B**. Representative examples comparing mitosis in control cells and cells treated with 20 μM PS-I. Still images from videomicroscopy are shown, with times indicated on the left. A full video is shown in Supplementary S3. **C**. Quantitation of the number of mitotic events observed during live cell image analysis (17 h) for U87MG cells control treated or treated with 20 μM PS-I. Data are the mean +/- SD of three independent videomicroscopy experiments for each condition. Inset table indicates the number of normal mitotic events (total starting cell numbers from the three experiments in parentheses) and the number of abnormal (delayed) mitotic events observed over the 17 h analysis for control treated and PS-I treated cells.

Similar to the observations in U87MG cells expressing shPKCιA, treatment of U87MG cells with the PS-I impaired cell proliferation (Figure 7A). Time-lapse videomicroscopy showed that this was also a consequence of impaired mitosis (Figure 7B, C and Additional file 4, Video 3). The mean number of mitotic events observed in three independent control experiments was 9 over 17 h (Figure 7C inset table). The total number of mitotic events observed in the three control experiments was 27, all of which where characterized as normal mitotic events based on timing (Figure 7C inset table). U87MG cells treated with PS-I had a significant reduction in the number of mitotic events: a mean of 3 was observed per 17 h video as compared to a mean of 9 in control videos. A total of 5 delayed mitotic events and 7 normal events were observed in the three independent live cell imaging analysis of PS-I treated U87MG cells (Figure 7C inset table). Similar impairments in mitosis were observed in videomicroscopy analyses of A172 cells in which PKCι was either depleted with shRNA or inhibited with PS-I (Additional file 5, Figure S2C).

## Discussion

We previously showed that PKCι promotes glioblastoma cell invasion [16]. This was primarily due to the ability of PKCι to promote cell motility and was linked to repression of RhoB expression by PKCι. To extend these findings, we have used time lapse videomicroscopy to characterize the motility defects in PKCι-depleted glioblastoma cells. PKCι-depleted cells actively extended multiple short protrusions, but have a markedly reduced ability to form a single leading edge lamellipodium, an essential feature of productive cell movement. This is consistent with the established role of the atypical PKCs in generating cell polarity in multiple contexts, including apical/basolateral polarity and asymmetric cell division. Our work is also consistent with the work of Etienne-Manneville *et al*., which showed the presence of an atypical PKC at the leading edge of migrating astrocytes in association with Cdc42, although in their study this was ascribed to PKCζ rather than PKCι [23].

**Figure 8:**
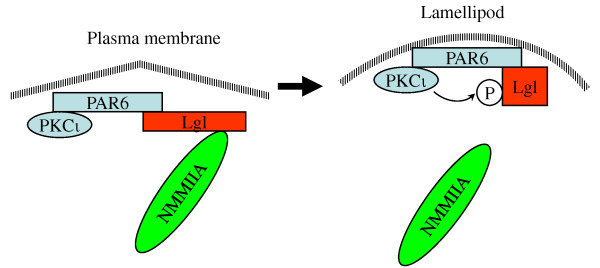
**Model for the role of PKCι in lamellipod formation**. An initial complex of the scaffolding protein Par6, PKCι and Lgl forms at the plasma membrane. Lgl is in its active form and binds non-muscle myosin IIA. PKCι then phosphorylates and inactivates Lgl. Uncoupling of Lgl from non-muscle myosin IIA then allows the actin remodeling required for lamellipod formation. In the absence of Lgl inactivation by PKCι, lamellipod formation is aborted and cells attempt to form lamellipodia at alternate sites.

The impaired motility of PKCι was associated with altered behavior of the cytoskeletal protein Lgl. Lgl was first described as a tumor suppressor in *Drosophila*, and has been shown to be a direct substrate for PKCι in both *Drosophila *and mammalian cells [19,20]. Phosphorylation of the central hinge region of Lgl inactivates the protein with respect to its non-muscle myosin II and membrane association functions [21,24]. Transduced Lgl was concentrated at the leading edge of glioblastoma cells in agreement with a previous report [20] and was constitutively phosphorylated in glioblastoma cells. Lgl was not associated with non-muscle myosin IIA, which was behind the lamellipod leading edge as described previously [22]. PKCι depletion reduced Lgl phosphorylation and changed its intracellular distribution such that it was concentrated at multiple sites around the periphery of the cell, where it colocalized with non-muscle myosin II. This is consistent with the videomicroscopy evidence that these cells initiate multiple uncoordinated attempts at leading edge formation. Figure 8 shows a model for the role of PKCι in lamellipod formation. PKCι is initially recruited to the plasma membrane as a complex with the scaffolding protein Par6 and Lgl [20]. At this point Lgl is associated with non-muscle myosin II via its carboxy terminal domain [21]. PKCι is then activated locally by PI 3-kinase and Cdc42. This inactivates Lgl, disrupting its association with non-muscle myosin II-containing actin filaments. This then allows actin remodeling and forward spreading of the lamellipod. In the absence of functional PKCι, lamellipod formation is aborted due to failed uncoupling of Lgl from non-muscle myosin II. Cells then attempt to form lamellipodia at other sites, which manifest as the multiple small protrusions observed by videomicroscopy. This model may also explain the previous finding that non-muscle myosin IIA inhibits cell motility [22]. The PKCι-mediated uncoupling of Lgl from non-muscle myosin II is a second mechanism by which PKCι can regulate actin dynamics, as we have previously shown that PKCι negatively regulates the expression of RhoB in glioblastoma cells and can influence actin dynamics in this manner [16]. These two mechanisms may act in concert, with RhoB repression leading to destabilization of actin filaments and Lgl/non-muscle myosin IIA uncoupling permitting forward spreading of the lamellipod.

Depletion or inhibition of PKCι in glioblastoma cells caused a marked decrease in cell proliferation under normal tissue culture conditions. This is in contrast to findings in other cancer types, where PKCι only affected anchorage-independent proliferation [10,25] and suggests a unique role for PKCι in glioblastoma. Videomicroscopy showed that the impaired proliferation was due, at least in part, to an impairment in mitosis. This was seen when PKCι levels were depleted by stable expression of a shRNA, or when PKCι activity was reduced using a selective inhibitor. PKCι has not been shown to have a role in mitosis previously. However, Wirtz-Peitz *et al*. have shown that in *Drosophila *atypical PKC is activated by Aurora-A kinase during mitosis and linked this activation to the establishment of asymmetric cell division in *Drosophila *neural precursors [26]. Our work shows a more direct role for atypical PKC in mitosis itself. Glioblastoma cells with reduced PKCι activity appeared to have two mitosis-related defects: (1) a reduction in cells entering mitosis; (2) a delay or failure to progress through mitosis normally. Aurora-A also has roles at multiple points during mitosis [27]. This parallel aspect to the behavior of the two kinases suggests that PKCι may be a downstream mediator of Aurora-A in mitosis. It will be important to determine if Aurora-A is in fact responsible for PKCι activation in this context. Some or all of the effects of PKCι on glioblastoma cell proliferation could be mediated by inactivation of Lgl. In both *Drosophila *and mice, mutational inactivation of Lgl not only causes polarity defects, but also induces uncontrolled proliferation in neural tissue [28,29]. A role for Lgl inactivation in glioblastoma proliferation would explain our observation that we could not isolate a stable population of glioblastoma cells expressing a non-phosphorylatable version of Lgl that cannot be inactivated by PKCι.

Our data indicate show a role for PKCι in both glioblastoma cell motility and mitosis. Cell movement and mitosis are mutually exclusive events: cells arrest movement and decrease their matrix attachments prior to mitosis. It is possible that these two processes involve separate intracellular pools of PKCι. Alternatively, a limited pool of PKCι might be co-opted away from motility functions during mitosis, contributing to the uncoupling of these two processes.

## Conclusions

PKCι promotes glioblastoma cell invasion by coordinating lamellipod leading edge formation and has a role in remodeling the cytoskeleton at the lamellipod leading edge, promoting the dissociation of Lgl from non-muscle myosin II. In addition PKCι is required for progression through mitosis in glioblastoma cells. The data presented here, along with our previously published data [16,18], show that PKCι has a role in multiple aspects of glioblastoma cell malignancy. These include the repression of apoptosis in response to DNA damage, aberrant proliferation and metastasis. PKCι is activated by several different oncogenic mutations in glioblastoma, and appears to have a non-redundant role in mediating signaling downstream of these mutations. These features suggest that PKCι is a promising target for glioblastoma therapy that warrants further investigation.

## Materials and methods

### Chemicals and antibodies

Antibodies to phospho-PKCι T555 and total PKCι were from BD Biosciences (Mississauga, ON, Canada). Mouse anti-Flag M2 antibody and rabbit non-muscle myosin IIA antibody were from Sigma (Oakville, ON, Canada). Antibody to PKC consensus phosphorylation site was from Cell Signaling Technology (Beverly, MA, USA). Secondary antibodies Alexa-Fluor 488 chicken anti-rabbit and Alexa Fluor 555 goat anti-mouse were from Invitrogen (Burlington, ON, Canada). Myristoylated atypical PKC pseudosubstrate peptide was from Invitrogen (Burlington, ON, Canada).

### Cell lines

The human glioblastoma cell line U87MG was obtained from Dr. W. Cavenee (Ludwig Institute for Cancer Research, La Jolla, CA). A172 cells were from the American Type Culture Collection. Cells were cultured at 37°C and 5% CO_2 _in Dulbecco's modified Eagle's medium (DMEM) supplemented with 100 units/ml penicillin, 100 μg/ml streptomycin, 2 mM glutamine and 10% (v/v) of a 2:1 mixture of donor bovine serum and fetal bovine serum. To make glioblastoma cell lines stably depleted of PKCι, short hairpin DNA target sequences (see Additional file 1, Figure S1A) were designed and ordered from Integrated DNA Technologies (Coralville, IA, USA). Sense and antisense strands were annealed and subcloned into the pSUPER.retro.puro backbone (OligoEngine, Seattle, WA, USA). Replication-incompetent retroviruses containing pshGFP, pshPKCιA or pshPKCιB were made as described previously [30]. Cells were gown in media containing puromycin (1 μg/mL) to select for transductants. Control retroviruses contained empty vector or shRNA to green fluorescent protein. Cells were used within three weeks of selection because of their marked growth impairment, which over time selected for cells with reduced PKCι depletion. To make U87MG cells expressing Flag-tagged Lgl, full-length *LLGL1 *cDNA (exact match with GenBank accession number NM_004140) was cloned from normal human astrocyte mRNA and subcloned into the retroviral vector pLPCX (Clontech, Palo Alto, CA. USA). Site-directed mutagenesis was then used to add an amino-terminal Flag epitope. Flag-tagged Lgl was expressed in U87MG cells using retroviral transduction followed by puromycin selection. Transient depletion of PKCι by RNA interference was done as described previously [18]; in some experiments an additional RNA duplex (designated C) was used that has been described previously [17].

### Western blot analysis

Western blotting was performed as described previously [18]. After electrophoretic transfer from the gel, blots were stained with amido black to confirm that equal sample loading and transfer was achieved.

### RT-PCR

RNA was isolated using the Qiagen's RNeasy Plus Mini Kit and cDNA was generated using the Qiagen Quantitect RT Kit. PCRs were then performed using the following primers: for PUM1 (reference gene), 5'TGAGGTGTGCACCATGAAC 3' and 5' CAGAATGTGCTTGCCATAGGG 3'; for PKCι, 5'GTCCGGGTGAAAGCCTACTAC 3' and 5'ACGGGTCTCCTTCCTCATCT 3'; for PKCζ 5'CCAAGAGCCTCCAGTAGACG 3' and 5'CCATCCATCCCATCGATAAC 3'.

### Cell counts

Live cell number was determined using a Vi-Cell XR cell viability analyzer with trypan blue exclusion (Beckman Coulter Canada Inc., Mississauga, ON, Canada).

### Cell motility and invasion assays

Chambers (BD Biocoat Matrigel invasion chambers, BD Biosciences, Mississauga, ON, Canada) were rehydrated and equilibrated for 2 h with 500 μL of serum free DMEM medium. After 2 h, the medium in the inserts was aspirated and inserts were placed into the wells containing complete DMEM (10% FBS:DBS). Chambers that were not coated with Matrigel (control inserts) were used to measure motility. Each chamber contains a membrane with 8 μm pores. U87MG stably expressing PKCι short hairpin were counted and resuspended in serum free DMEM medium at 1 × 10^5 ^cells/ml. Five hundred μl of cell suspension (50 000 cells) were added to each chamber. The chambers were incubated for 22 h at 37°C in a 5% CO_2 _atmosphere. The media was then removed and the upper surface of the membrane was scrubbed ten times with a cotton swab. Cells on the lower surface of the scrubbed membranes were fixed in 10% methanol and stained with Diff-Kwik (Dade-Behring, Newark, DE) according to the manufacturer's instructions. Three random fields were counted from each chamber under the light microscope at 40× magnification.

### Time lapse videomicroscopy

Control and PKCi depleted or pseudosubstrate treated U87MG cells were plated into a Bioptechs Delta T (Butler, PA) live cell imaging plate in 2 mL of complete DMEM at a density of 10^3 ^cells to allow space for migration. Cells were maintained at 37°C in 5% CO_2 _for the duration of the videos. Videomicroscopy was done using an inverted microscope (Ziess Axiovert 200 M) equipped with phase-contrast microscopy using a 10× objective. Images were acquired with a CCD camera (AxioCam HRm) driven by Zeiss Axiovision 4.5 software. Phase contrast images of the cells were taken at 5 min intervals for 17-20 h and compiled to generate a time-lapse video. To quantify the migration distance per minute, cells from three independent time-lapse imaging experiments of each cell line were analyzed using the Zeiss LSM image browser software. Cell nuclei were tracked to determine migration distance and divided by the travel time. To quantify leading edges, cells from independent movies were assessed for the formation of a single dominant leading edge. Once a cell generated and formed a single leading edge it was counted. A cell was only counted once throughout the duration of the movie (*i.e*. if it changed direction and generated a new leading edge it was not counted a second time).

### Immunofluorescence and confocal microscopy

Cells were grown in 6-well TC dishes containing glass coverslips pre-coated 0.15% Gelatin. They were washed briefly in cold (4°C) PBS and fixed in cold 4% paraformadehyde for 30 minutes. Next, cells were washed in PBS 3 times for 5 minutes each, permeablized for 10 minutes in 0.2% Triton-X 100 (diluted in PBS), and washed in PBS again 3 times for 5 minutes each. Cells were blocked in a solution made of 5% normal goat serum and 5% normal chicken serum in PBS for 30 minutes at room temperature. Cells were then incubated for 1 hour at RT with the primary antibody cocktail which consisted of 1 ug/ml Mouse anti-Flag M2 antibody and 1:200 dilution of rabbit non-muscle myosin IIA diluted in the blocking solution. Cells were washed gently 3 times for 10 minutes each in PBS. This was followed by a 45 minute at RT incubation with the secondary antibody cocktail consisting of 2 ug/ml each of Goat anti-Mouse Alexa Fluor 555 and 2 ug/ml Chicken anti-Rabbit Alexa-Fluor 488 diluted in the blocking solution. Finally the cells were washed with three 10 minutes washes in PBS, mounted on a glass slide with Prolong gold with DAPI (Invitrogen Cat # P-36931) and allowed to air dry overnight at RT in the dark. The following day, coverslips were sealed with permanent mounting media (DAKO Cat. # S3026).

Fluorescent labeling was observed using a Zeiss Observer. Z1 microscope (63X/1.40 oil DIC M27 objective) connected to a Zeiss LSM 510 Meta confocal unit. Alexa Fluor 555 was excited using the He-Ne 543 laser set at 50% power and channeled through an HFT 488/548 main dichroic filter, an NFT 545 secondary dichroic filter and a BP 560-615 IR. Images were captured using Zeiss' ZEN (version 4.5) software for the confocal microscope. AF 488 was excited with the Argon laser set at 20% power and channeled through an HFT 488/548 main dichroic filter and a BP 505-530 filter. DAPI was excited using the He-Ne 405 laser set at 20% power channeled through an HFT 405/488 main dichroic filter and a BP 420-480 filter. Image stacks were collected with the software Pinhole set at 1 Airy unit and a slice interval of 0.41 um.

### Statistical analysis

All results were expressed as the mean ± S.D. Statistical analysis was performed using the Student's *t *test. P < 0.05 was considered statistically significant and is indicated by the symbol *.

## Declaration of Competing interests

The authors declare that they have no competing interests.

## Authors' contributions

RMB carried out the videomicroscopy analyses and drafted the manuscript. GMB initiated the experiments on Lgl and contributed to the design of the study. DAEP performed the immunofluorescence microscopy experiments. JKG performed the experiments on Lgl phosphorylation. JAP and SJL performed the videomicroscopy analyses of A172 cells. DAG assisted with the videomicroscopy experiments. IAJL conceived of the study, and participated in its design and coordination and prepared the final manuscript. All authors read and approved the final manuscript.

## Supplementary Material

Additional file 1 **Figure S1. shRNA sequences and atypical PKC isoform expression in glioblastoma cells**. **A**. shRNA encoding sequences ligated into the pSUPER.retro.puro shRNA expression plasmid. **B**. PKCι and PKCζ mRNA expression was assessed in U87MG and A172 human glioblastoma cells by RT-PCR. The human breast cancer cell line was used as a positive control for PKCζ expression and Pum1 mRNA was assessed as a control to show that the input of mRNA and cDNA in each reaction was similar. Click here for file

Additional file 2 **Video 1. Time lapse videomicroscopy of U87MG cells**. The video shown is of U87MG cells that were transduced with retrovirus made with pLPCX vector expressing a shRNA to green fluorescent protein (as a control). Time lapse videomicroscopy was performed as described in Materials and Methods. Images were taken at 5 min intervals for 20 h. The video is a representative example of three videos of these cells. Click here for file

Additional file 3 **Video 2. Time lapse videomicroscopy of U87MG cells expressing PKCι shRNA**. The video shown is of U87MG cells that were transduced with retrovirus expressing PKCι shRNA. Other conditions were as in Additional file 2. The video is a representative example of three videos of these cells. Click here for file

Additional file 4 **Video 3. Time lapse videomicroscopy of U87MG cells treated with PKCι pseudosubstrate inhibitor peptide**. The video shown is of U87MG cells treated with 20 μM pseudosubstrate peptide. Other conditions were as in Additional file 2. The video is a representative example of three videos of these cells. Click here for file

Additional file 5 **Figure S2. Motility, proliferation and mitosis in A172 glioblastoma cells depleted of PKCι**. **A**. *Quantitation of A172 motility from videomicroscopy*. Migration distance per minute (um/min) was measured using Ziess LSM image browser software. Bars show the mean +/- SD from three independent videomicroscopy experiments for A172 cells and A172 cells stably depleted of PKCι and the mean ± range from two independent experiments for A172 cells treated with 20 uM pseudosubstrate inhibitor peptide (PS-I). **B**. *Effects of transient PKCι depletion on U87MG and A172 proliferation*. U87MG cells (left panel) and A172 cells (right panel) were either mock-transfected, transiently transfected with a control RNA duplex, or transiently transfected with two different duplexes targeting PKCι. Viable cell numbers were determined using trypan blue exclusion on the indicated days after transfection. **C**. *Quantitation of A712 mitoses from videomicroscopy*. Data are from three independent movies for A172 and A172/pshPKCιA cells and two independent movies for A172 cells treated with 20 uM PS-I. Click here for file
