# Supplementary material for: Coordination of glioblastoma cell motility by PKCι
Source: Mol Cancer. 2010 Sep 3;9:233. doi: 10.1186/1476-4598-9-233 (PMC2941485; doi:10.1186/1476-4598-9-233)

A.

Sense — Hairpin — Antisense

shGFP: 5’ *GATCCCC***AAGCTGGAGTACAACTACA***TTCAAGAGA***TGTAGTTGTACTCCAGCTT** *TTTTTA*

shPKCιA: 5’ *GATCCCC***GTGCATCAACTGCAAACTC***TTCAAGAGA***GAGTTTGCAGTTGATGCAC***TTTTTA*

shPKCιB: 5’ *GATCCCC***TGAGGTTCGAGACATGTGT***TTCAAGAGA***ACACATGTCTCGAACCTCA***TTTTTA*

B.

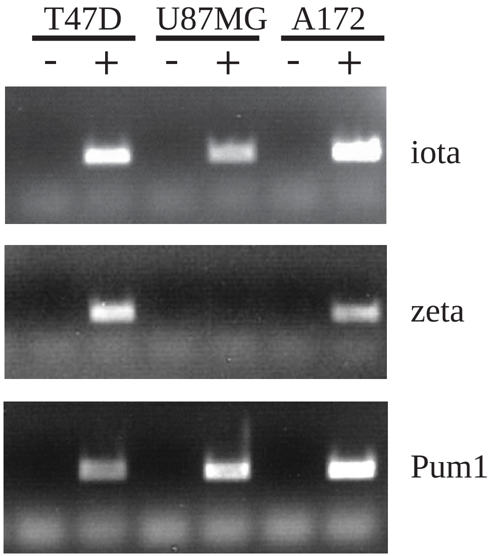

Supplement: Additional file 1 — Figure S1. shRNA sequences and atypical PKC isoform expression in glioblastoma cells. A. shRNA encoding sequences ligated into the pSUPER.retro.puro shRNA expression plasmid. B. PKCι and PKCζ mRNA expression was assessed in U87MG and A172 human glioblastoma cells by RT-PCR. The human breast cancer cell line was used as a positive control for PKCζ expression and Pum1 mRNA was assessed as a control to show that the input of mRNA and cDNA in each reaction was similar. [file 1476-4598-9-233-S1.PDF]
