# Supplementary figures and images for: Coordination of glioblastoma cell motility by PKCι
Source: Mol Cancer. 2010 Sep 3;9:233. doi: 10.1186/1476-4598-9-233 (PMC2941485; doi:10.1186/1476-4598-9-233)

A.

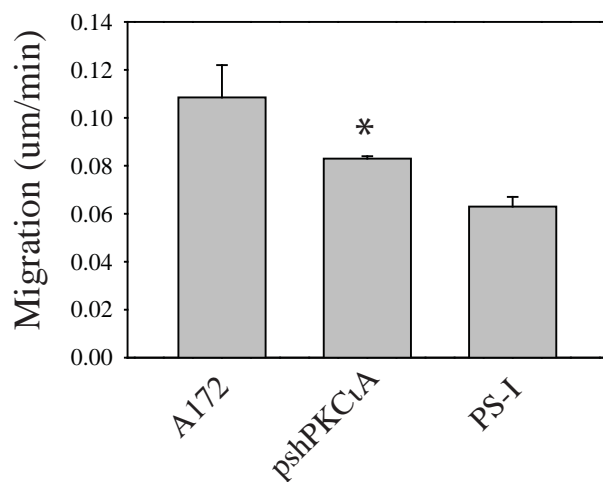

B.

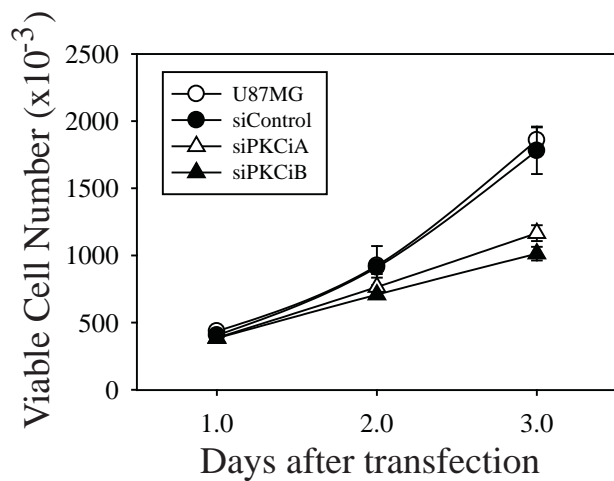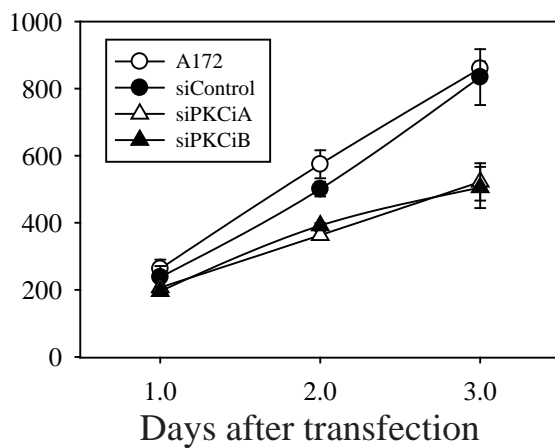

C.

|          | Normal Mitotic Events | Total Initial Cell Number | Percent |
|----------|-----------------------|---------------------------|---------|
| A172     | 8                     | 61                        | 13      |
| pshPKCtA | 0                     | 54                        | 0       |
| PS-I     | 1                     | 36                        | 3       |

Supplement: Additional file 5 — Figure S2. Motility, proliferation and mitosis in A172 glioblastoma cells depleted of PKCι. A. Quantitation of A172 motility from videomicroscopy. Migration distance per minute (um/min) was measured using Ziess LSM image browser software. Bars show the mean +/- SD from three independent videomicroscopy experiments for A172 cells and A172 cells stably depleted of PKCι and the mean ± range from two independent experiments for A172 cells treated with 20 uM pseudosubstrate inhibitor peptide (PS-I). B. Effects of transient PKCι depletion on U87MG and A172 proliferation. U87MG cells (left panel) and A172 cells (right panel) were either mock-transfected, transiently transfected with a control RNA duplex, or transiently transfected with two different duplexes targeting PKCι. Viable cell numbers were determined using trypan blue exclusion on the indicated days after transfection. C. Quantitation of A712 mitoses from videomicroscopy. Data are from three independent movies for A172 and A172/pshPKCιA cells and two independent movies for A172 cells treated with 20 uM PS-I. [file 1476-4598-9-233-S5.PDF]
